# Cost–benefit analysis of the polypill in the primary prevention of myocardial infarction and stroke

**DOI:** 10.1007/s10654-016-0122-1

**Published:** 2016-03-05

**Authors:** Nicholas J. Wald, Johannes Michiel Luteijn, Joan K. Morris, David Taylor, Peter Oppenheimer

**Affiliations:** Wolfson Institute of Preventive Medicine, Barts and the London School of Medicine and Dentistry, Queen Mary University of London, London, EC1M 6BQ UK; UCL School of Pharmacy, BMA/Tavistock House, London, WC1H 9JP UK; Christ Church, Oxford, OX1 1DP UK

**Keywords:** Cost–benefit analysis, Polypill, Primary prevention, Cardiovascular diseases, Stroke, Myocardial infarction

## Abstract

The primary prevention of cardiovascular disease is a public health priority. To assess the costs and benefits of a Polypill Prevention Programme using a daily 4-component polypill from age 50 in the UK, we determined the life years gained without a first myocardial infarction (MI) or stroke, together with the total service cost (or saving) and the net cost (or saving) per year of life gained without a first MI or stroke. This was estimated on the basis of a 50 % uptake and a previously published 83 % treatment adherence. The total years of life gained without a first MI or stroke in a mature programme is 990,000 each year in the UK. If the cost of the Polypill Prevention Programme were £1 per person per day, the total cost would be £4.76 bn and, given the savings (at 2014 prices) of £2.65 bn arising from the disease prevented, there would be a net cost of £2.11 bn representing a net cost per year of life gained without a first MI or stroke of £2120. The results are robust to sensitivity analyses. A national Polypill Prevention Programme would have a substantial effect in preventing MIs and strokes and be cost-effective.

## Introduction

Cardiovascular disease, particularly myocardial infarction (MI) and stroke, is one of the leading causes of death and disability throughout the world. This is so even in countries such as the USA and United Kingdom (UK) where age specific mortality rates from MI and stroke have declined [1, 2].

It is recognised that the primary prevention of cardiovascular disease is important. To this end, recommendations that people should reduce their salt, sugar and saturated fat intake, take regular exercise, control their weight, and avoid smoking, are widely accepted. In addition it is generally accepted that people at sufficiently high risk of an MI or stroke should be identified so that they can receive preventive medication. This medication could be in the form of a combination pill (polypill) consisting of a statin to lower LDL cholesterol, and low dose blood pressure lowering drugs to reduce blood pressure [3–6]. It has been established, on the basis of epidemiological evidence and from randomised trials, that reducing these risk factors has a substantial impact in reducing the risk of MIs and strokes [6, 7].

The results presented in a previous paper [8, 9] are intended to guide individuals considering participation in a Polypill Prevention Programme by showing that one in three people who take the polypill will benefit and gain, on average, 8 years of life without a first MI or stroke. The present paper is intended to produce results to guide policymakers considering setting up a Polypill Prevention Programme as a public service. We assess the economic implications of the polypill approach with particular reference to its possible adoption by the UK National Health Service. The results may be relevant to other similar health services throughout the world. Our aim was to determine (i) the total number of years of life gained without a first MI or stroke (years in a group offered the polypill minus years in an identical group not offered the polypill), (ii) the total annual cost, and (iii) the cost (or saving) per year of life gained in this way from a Polypill Prevention Programme for individuals aged 50 and over in the UK.

## Methods

We performed a standard life table analysis based on methods and results published in Wald and Morris [8, 9] to determine the number of MIs and strokes and the years of life gained without a first MI or stroke from delivering a public health prevention programme based on a four component polypill consisting of 20 mg simvastatin, 2.5 mg amlodipine, 25 mg losartan and 12.5 mg hydrochlorothiazide (recognising that, in the future, alternative formulations may be advantageous). Briefly, life tables started with the 453,913 males and 465,472 females aged 50 in the UK in 2013 [10] by applying annual age- and sex-specific risks of a first MI, first stroke and death from causes other than MI and stroke separately to create two cohorts of people aged 50 in the UK in 2013: one cohort taking the polypill and the other not taking the polypill. At the end of each year of age a person could be: (i) alive without ever having had an MI or stroke, or (ii) alive or dead having had an MI or stroke, or (iii) dead without ever having had an MI or stroke. Over time, individuals can move from 1 to 2 or from 1 to 3, but not from 2 to 3, 2 to 1, or 3 to 1. For people who did not take the polypill, the probability of moving from state 1 to 2 was the age-sex specific annual incidence of the first occurrence of an MI or stroke, and the probability of moving from state 1 to 3 was the age-sex specific annual mortality from all causes, excluding MI or stroke. For people taking the polypill, the probability of moving from state 1 to 2 was the age-sex specific annual incidence of the first occurrence of an MI or stroke multiplied by the age-sex specific relative risk reductions from taking the polypill, and the probability of moving from state 1 to 3 was the same as for people who did not take the polypill (see “Appendix” for the derivation of the probabilities used). The survival times and number of first MIs and strokes were accumulated. The costs were obtained by multiplying the survival times by the costs of taking the polypill daily and the total costs of first MIs by the number of first MIs multiplied by the average cost of treating an MI and the total costs of first strokes by the number of first strokes multiplied by the average cost of treating a stroke. The average cost of treating an MI was taken from Luengo-Fernandez et al. [11] and the average cost of treating a stroke was taken from Saka et al. [12]. The costs of treating an MI and stroke were adjusted to the cost in 2014 using the UK Treasury inflation figures [13]. Details of how the costs from these papers were taken are given in the “Appendix”.

The non-discounted lifetime cost, saving on treatment and years of life gained without a first MI or stroke from implementing a Polypill Prevention Programme in a cohort of people aged 50 were estimated. If the size of each annual cohort of people aged 50 is constant over time, these estimates are equivalent to annual figures for a mature programme. The programme becomes mature after about 20 years when there is a balance in the number of people in two groups: (i) the annual number who have an MI or stroke prevented and (ii) the annual number who die (from any cause) or have a non-fatal MI or stroke among everyone who had an MI or stroke prevented at any time in the past. Then the programme is in a steady state in which it is necessary to compare only the constant annual cost of the programme with the constant annual monetary value of the benefit.

Our estimates of the preventive effects of the polypill relate only to the incidence of MI and stroke. The estimate of 17 % for non-adherence was taken from experience of a Polypill Prevention Programme using the separate polypill drug components [14]. Non-adherent individuals were modelled to participate in a Polypill Prevention Programme for 1 month, without experiencing any health benefits, before dropping out of the programme and not involving any further cost.

The prescription, dispensing, distribution and manufacturing of the polypill was considered at several cost levels per (daily) pill, ranging from £0.50 to £1.50. A private Polypill Prevention Programme is already available online (polypill.com) at a cost per polypill of £1.05, which includes the complete cost of delivering the service. We separately allocated a £5 one-off cost per person invited to join the programme, to cover invitation and programme start-up expenses. A summary of these unit costs is set out in Table 1.

**Table 1:** Input estimates used in the analysis

Item	Value
Program invitation per person invited (one-off invitation letter and infrastructure cost)	£5
Cost of providing polypill (per person per day)^c^	£0.50, 0.75, 1.00, 1.25 and 1.50
Average healthcare cost of an MI (per clinical event)^c^	£29,900 [11, 15]
Average healthcare cost of a stroke (per clinical event)^c^	£50,500 [12]
Polypill uptake	50 %
Polypill adherence	83 % [14]
Polypill LDL cholesterol reduction^a^	1.54 mmol/L [5]
Polypill diastolic BP reduction^a^	10.7 mmHg [6]
Age-specific relative risk for MI on polypill at age 60^b^	0.23 [5, 16]
Age-specific relative risk for stroke on polypill at age 60^b^	0.28 [5, 6, 16]

^a^Simvastatin 20 mg, amlodipine 2.5 mg, hydrochlorothiazide 12.5 mg, losartan 25 mg

^b^Age-specific relative risks from age 50 were applied in the model

^c^At 2014 prices

The annual years of life gained without a first MI or stroke arising from a Polypill Prevention Programme was estimated, as well as: (i) annual health service saving arising from the reduction in MI or stroke events; (ii) annual health service cost of the Polypill Prevention Programme; (iii) net annual health service cost (or saving) arising from (i) and (ii); and (iv) net cost (or saving) per MI or stroke prevented. These estimations were performed for different costs of providing a daily polypill. A hypothetical “best case” situation with 100 % uptake and 100 % adherence and a “working case” situation with 50 % uptake of the polypill and 83 % adherence were considered.

We also carried out sensitivity analyses by varying the key input variables in turn by ±25 %.

## Results

Table 2 shows the total annual years of life gained without a first MI or stroke in the UK in a hypothetical best case in which the polypill uptake and adherence rates are both 100 % and in a more realistic working case with a 50 % uptake and 83 % adherence (2,390,000 and 990,000 years respectively). The table also shows the extra cost or saving (i.e. net cost or saving) per year of life gained without a first MI or stroke according to the daily per person cost of the Polypill Prevention Programme. For example, the net cost per year of life gained without a first MI or stroke would be £2120 if the daily cost of a Polypill Prevention Programme was £1 per person.

**Table 2:** Total years of life gained and net costs (at 2014 prices) per year of life gained without a first myocardial infarction (MI) or stroke in people aged 50 and over in a UK Polypill Prevention Programme

	Total years of life gained without a first MI or stroke in the UK (thousand)	Daily cost of Polypill Prevention Programme per person (£)				
		0.50	0.75	1.00	1.25	1.50
		Net cost or saving (–) per year of life gained without a first MI or stroke				
		(£)	(£)	(£)	(£)	(£)
*Best case*						
(100 % uptake, 100 % adherence)	2390	**−**280	920	2120	3310	4510
*Working case*						
(50 % uptake, 83 % adherence)	990	**−**270	920	2120	3320	4520

Table 3 shows the total annual health service savings made through preventing MI and strokes in a Polypill Prevention Programme (£6.39 and £2.65 bn for the best case and working cases respectively). The table also shows, according to the specified daily per person cost of the programme, the total annual UK cost, and the net annual UK cost. For example, if the daily cost of the programme were £1 per person, the total annual cost in the working case would be £4.76 bn and the net annual cost would be £2.11 bn (£4.76–£2.65 bn). The cost estimates for each of the four countries in the UK are shown in the “Appendix” based on their populations [10]. The total years of life gained without a first MI or stroke are 828,000, 89,000, 47,000 and 28,000 respectively for England, Scotland, Wales and Northern Ireland.

**Table 3:** Total saving, total cost and net cost or saving each year (at 2014 prices) in people aged 50 and over in a UK Polypill Prevention Programme

	Total saving from reducing incidence of MI and stroke (£bn)	Daily cost of Polypill Prevention Programme per person (£)									
		0.50		0.75		1.00		1.25		1.50	
		Total cost of polypill prevention programme (£bn)	Net cost or saving (£bn)	Total cost of polypill prevention programme (£bn)	Net cost or saving (£bn)	Total cost of polypill prevention programme (£bn)	Net cost or saving (£bn)	Total cost of polypill prevention programme (£bn)	Net cost or saving (£bn)	Total cost of polypill prevention programme (£bn)	Net cost or saving (£bn)
	(1)	(2)	(2–1)	(3)	(3–1)	(4)	(4–1)	(5)	(5–1)	(6)	(6–1)
*Best case*											
(100 % uptake, 100 % adherence)	6.39	5.73	−0.66	8.59	2.20	11.45	5.06	14.31	7.92	17.17	10.78
*Working case*											
(50 % uptake, 83 % adherence)	2.65	2.38	−0.27	3.57	0.92	4.76	2.11	5.94	3.29	7.13	4.48

Table 3 also shows that if the cost of providing the polypill were relatively low (£0.50 per person per day) there would be a net saving per year of life gained without a first MI or stroke. As the cost increases, the net saving per year of life gained gradually disappears and turns into a net cost. For example, if the cost per person per day were £1.50 the net cost per year of life gained without a first MI or stroke would be £4520 (Table 2). If the cost per person per day were £0.56, a Polypill Prevention Programme would be cost neutral.

Tables 4 and 5 show the effect on the working case of altering in turn four key input estimates (incidence of MI and stroke, polypill efficacy, NHS cost per MI and stroke, and non-adherence) by setting these input estimates at 25 % less than those used in the model and at 25 % more, thus providing an indication of how such variation influences the results (i.e. a sensitivity analysis). This sensitivity analysis shows that none of the alterations affects the cost per year of life gained without a first MI or stroke by more than about ±£1000.

**Table 4:** Sensitivity analyses relating to results shown in Table 2 for the “working case”

	Total years of life gained without a first MI or stroke in the UK (thousand)	Daily cost of Polypill Prevention Programme per person (£)				
		0.50	0.75	1.00	1.25	1.50
		Net cost or saving per year of life gained without a first MI or stroke				
		(£)	(£)	(£)	(£)	(£)
*Incidence of MI and stroke*						
25 % less	790	240	1760	3290	4820	6350
25 % more	1180	(530)	460	1460	2460	3460
*Polypill effectiveness*						
25 % less	800	330	1790	3260	4730	6190
25 % more	1150	(640)	400	1440	2490	3530
*Cost of MI and stroke*						
25 % less	990	390	1590	2790	3990	5190
25 % more	990	(940)	250	1450	2650	3850
*Non-adherence to treatment*						
25 % less	1040	(280)	920	2120	3320	4520
25 % more	940	(270)	920	2120	3320	4520

**Table 5:** Sensitivity analyses relating to results shown in Table 3 for the “working case”

	Total saving from reducing incidence of MI and stroke (£bn)	Daily cost of Polypill Prevention Programme per person (£)									
		0.50		0.75		1.00		1.25		1.50	
		Total cost of Polypill Prevention Programme (£bn)	Net cost or saving (£bn)	Total cost of Polypill Prevention Programme (£bn)	Net cost or saving (£bn)	Total cost of Polypill Prevention Programme (£bn)	Net cost or saving (£bn)	Total cost of Polypill Prevention Programme (£bn)	Net cost or saving (£bn)	Total cost of Polypill Prevention Programme (£bn)	Net cost or saving (£bn)
	(1)	(2)	(2–1)	(3)	(3–1)	(4)	(4–1)	(5)	(5–1)	(6)	(6–1)
*Incidence of MI and stroke*											
25 % less	2.22	2.41	0.18	3.61	1.39	4.81	2.59	6.01	3.79	7.21	4.99
25 % more	2.98	2.36	−0.63	3.53	0.55	4.71	1.73	5.88	2.90	7.06	4.08
*Polypill effectiveness*											
25 % less	2.09	2.09	0.26	3.53	1.44	4.71	2.61	5.88	3.79	7.06	4.96
25 % more	3.14	2.40	−0.74	3.60	0.46	4.80	1.66	6.00	2.86	7.20	4.06
*Cost of MI and stroke*											
25 % less	1.99	2.38	0.39	3.57	1.58	4.76	2.77	5.94	3.95	7.13	5.14
25 % more	3.32	2.38	−0.94	3.57	0.25	4.76	1.44	5.94	2.62	7.13	3.81
*Non-adherence to treatment*											
25 % less	2.79	2.50	−0.29	3.75	0.96	5.00	2.21	6.25	3.46	7.50	4.71
25 % more	2.52	2.26	−0.26	3.39	0.87	4.51	2.00	5.64	3.12	6.77	4.25

**Fig. 1:**
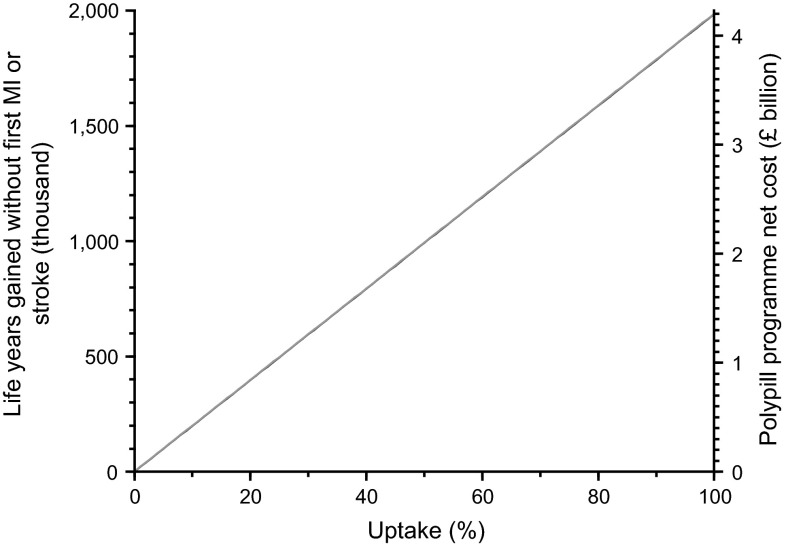
Annual life years gained without a first myocardial infarction or stroke and annual programme cost at 2014 prices for a Polypill Prevention Programme for individuals aged 50 and over according to uptake of polypill (£1 daily cost of providing a polypill, adherence to treatment 83 %)

Figure 1 shows the impact of polypill uptake on programme net cost and overall years of life gained without a first MI or stroke. Both increase with increasing uptake such that the cost per year of life gained without a first MI or stroke remains almost constant.

## Discussion

Our analysis shows that a Polypill Prevention Programme in which people aged 50 and over are offered a daily polypill would be effective in the prevention of MI and stroke. In our working case approximately one million years of life without a first MI or stroke in the UK would be gained every year. There are few public health programmes that could deliver such great gains in reducing morbidity and mortality in many countries throughout the world.

The cost of a mature Polypill Prevention Programme per year of life gained without a first MI or stroke would be approximately £2000 if the cost of providing the polypill were £1 per day per person and, in the working case, £2 billion annually for the UK as a whole (approximately 1.5 % of UK Public Sector Health Expenditure in 2014 [17]). Our analysis uses the concept of “years of life gained without a first MI or stroke” instead of simply “years of life gained”, because our focus is the prevention of non-fatal and fatal events. In some instances years of life gained may be the preferred measure of health benefit. An example is breast cancer screening where the method of prevention is early cancer detection and its treatment, in which years of life gained without being aware of breast cancer would be reduced. However in the primary prevention of a disorder that causes early death and morbidity, the preferred measure is life years gained without the disorder. Also, we deliberately did not adopt an adjusted measure known as “quality adjusted life years” or “QALYs”. Any adjustment due to disability arising from an MI or stroke would not be relevant in our analysis because we estimated years of life gained *without* either of these events. In any event, if we had estimated total life years gained, we would have been reluctant to use any adjustments which imply that the life of a disabled individual is of less value than that of a similar individual living without disability. It is increasingly recognised that the quality of life is a personal matter, and not one where the State or other agencies should impose their judgement [18, 19]. Notwithstanding these considerations, a cost of £2000 per year of life gained without a first MI or stroke is amply cost-effective in relation to the conventionally accepted maximum cost figure of between £20,000 and £30,000 per quality adjusted year of life gained allowed by NICE [20].

Our analysis combines data on males and females. The age-specific incidences being less in females, the cost per year of life gained without an MI or stroke is about £1000 more in females than males if the cost of delivering a polypill service is £1 per person per day. If the costs were set to be the same in both sexes the female cut-off would be about 10 years later (age 60) but, on average, women would lose about 8 months of extra life without an MI or stroke. The sex-specific policy trade-offs are finely balanced and do not justify separate age cut-offs for males and females [21].

The sensitivity analyses indicate that our estimates are robust to variations in the factors considered (Tables 4 and 5). For example, it is likely that a Polypill Prevention Programme will be taken up more readily among people in higher socio-economic groups. This will have only a modest effect on our estimates; even if the MI and stroke incidence were three times as great in lower socio-economic groups than in higher, and if twice as many people in higher socio-economic groups were to take up the polypill, the incidence of these disorders would be about 10 % lower in people taking the polypill than in the population as a whole, well within the limits of our sensitivity analysis on incidence.

Our results are not influenced by secondary prevention services because our paper is limited to the prevention of first events. The estimates are based on introducing a Polypill Prevention Programme in a population not receiving medication for primary prevention. At present in England there is a primary prevention programme provided by the National Health Service called “Health Checks” that involves adopting a Framingham-type screening approach. Although it might be argued that the polypill approach should be directly compared with this approach, there is both a scientific and a practical reason not to do so. The scientific reason is that provided the cost of a Polypill Prevention Programme is not excessive, it would be more cost effective, as shown in an earlier analysis comparing age screening with screening using multi-factor risk scores [22]. The practical reason is that the English Health Check programme lacks specificity and clarity over the interventions offered, and over the effect of these interventions in reducing morbidity and mortality, thereby making it impossible reliably to assess costs and benefits. The Polypill Programme overcomes these weaknesses and should be assessed independently of any other intervention.

The effect of a Polypill Prevention Programme on the total years of life gained without a first MI or stroke in the UK will depend on the size of the progressive annual cohorts of people aged 50, which can vary by up to 15 % from year to year. As a result, the total of such years gained is unlikely to fall below 850,000. This variation in the 50-year-olds cohort size does not, however, affect the cost per year gained, because as less people take up the polypill, the benefits and costs decline together almost pro rata.

The years of life gained without a first MI or stroke decline with increasing age of starting. For example, starting at age 55 instead of 50 in the working model, the gain in years of life without a first MI or stroke is reduced by about 10 % (910,000 instead of 990,000). At the same time, the net cost is reduced by about 30 % (£1.50 bn instead of £2.11 bn) and the cost per year of life gained without a first MI or stroke is reduced by about 20 % (£1650 instead of £2120). The disadvantage in setting a higher age cut-off, of course, is the failure to prevent MIs and strokes in younger people, and a reduction in overall public health benefit.

Reducing LDL cholesterol and blood pressure will prevent cardiovascular diseases other than MI and stroke, such as angina pectoris and aortic aneurysm. The Polypill Prevention Programme may also have a beneficial effect in preventing arteriosclerotic dementia. There is evidence that use of the polypill will reduce the prevalence of headaches by approximately one-third [23]. Most of the side effects of the polypill relate to intolerance and are not serious. This will result in some people deciding to stop taking it (in our example 17 %). The main serious side effect is rhabdomyolysis, arising from the use of statins, with an estimated risk of approximately 3 per 100,000 persons per year, and mortality of approximately 0.3 per 100,000 persons per year [24]. This estimate is consistent with the observation that in 2013 in England and Wales 7.6 million people took statins [25, 26] and 86 deaths were recorded as being due to rhabdomyolysis (ICD-10, M62.8) [27], of which about 20 would have been statin related. Statins increase the incidence of diabetes by an estimated 9 % but it is good practice to prescribe, in such cases, the components in the polypill, as the benefit far outweighs the risks [28].

Discounting the value of future health benefits and financial cost/saving in economic analyses of public health programmes is debatable [18, 29, 30], but it is irrelevant to our analysis. Once the programme is mature, there is a steady state between annual costs and benefits, both being constant from year to year, thus dispelling any possible rationale for discounting.

A challenge in introducing an NHS Polypill Prevention Programme will be to secure professional and public acceptance that the focus should be on providing effective and safe preventive treatment, rather than paying more attention to screening measurements [22]. Such measurements add little beyond the use of age to the prediction of cardiovascular disease. They do, however, add significantly to the workload of medical staff, arising from the associated extra medical consultations, laboratory tests, implementation of screening algorithms and risk counselling. Many doctors may feel that an individual should receive cholesterol lowering treatment only if the LDL cholesterol is raised or blood pressure lowering treatment only if his or her blood pressure is raised above essentially arbitrary cut-off values. This however means that some people at risk do not receive preventive treatment and others receive only some of the components in the polypill, when using all of them confers greater efficacy. Preventive treatment should involve reducing both LDL cholesterol and blood pressure, regardless of pre-treatment levels, because the benefit of doing so is not limited to people with high levels [16, 31, 32].

If everyone aged 50 and over in the UK were invited to join a Polypill Prevention Programme in 1 year, approximately 22 million people would be invited, representing about 2300 per GP practice in year one, and about a hundred in each year thereafter. If recruiting 2300 people in a single year poses too heavy an administrative burden on each practice (about 45 per week), recruitment could be phased over 2 years. That would mean about 1200 invitations per year in the first 2 years (about 22 per week) and about 100 per year thereafter. Perhaps more importantly, once a Polypill Prevention Programme were underway, there would be only about 100 new invitations each year per practice.

General Practice surgeries would identify people on their list when they reach their 50^th^ birthday, write to them to determine contraindications to preventive cardiovascular disease treatment (such as certain pre-existing diseases or medications which each person would indicate in a response to a short list of questions) and, if eligible, offer them a polypill to diminish the likelihood of future MIs and strokes. Acceptance could be done by mail or email and a prescription sent to a pharmacy for dispensing. The polypill could be sent to each polypill participant by post, or could be collected from a designated local pharmacy. The process could be implemented and monitored in a largely automated way, releasing General Practitioner time and resources for patients with medical problems.

Identifying people by age as being eligible for a polypill avoids them feeling that they are patients or being regarded as patients. They do not have a medical disorder that needs treatment; they choose to take a preventive medication to avoid becoming a patient. The UK National Health Service (NHS) and other collectively funded health care systems such as US Health Maintenance Organizations are ideal settings in which to implement the polypill concept.

Others who have conducted cost-effectiveness analyses, adopting different screening strategies and different estimates of cost, have concluded that cardiovascular disease prevention with a polypill is cost-effective, across a range of estimates of drug efficacy and treatment cost [33–35]. For example, an Australian study [35] ranked a polypill strategy as one of the most cost-effective interventions in the prevention of cardiovascular disease. Another study assessed an age-based screening strategy in low and middle income countries using an age cut-off of 55 years as being cost-effective [34]. In 2014 the US Rand Corporation conducted a case-study of “A Cardiovascular Polypill” [36] and again found it to be cost-effective [37].

Our analysis adds to the information available from previous economic analyses of the polypill. It focuses on three important measures relevant to assessing the merits of a National Polypill Prevention Programme: net cost for a total programme, years of life gained without a first MI or stroke, and net cost per year of life gained without a first MI or stroke. These estimates are here applied to the UK as a whole, to guide the National Health Service and other similarly managed health care services to develop policy in this area. The NHS could introduce a Polypill Prevention Programme generally or conduct a prior demonstration project in a large sample of GP practices within the UK, and audit the project.

From the perspective of each individual, and that of society as a whole, a Polypill Prevention Programme offers considerable health benefits at a relatively low cost.

## Appendix

Details of the data used in the life table analysis to determine the number of first myocardial infarctions (MIs) and strokes and the years of life gained without a first MI or stroke are given below:

1. The incidence of first myocardial infarction (MI) or stroke in England and Wales in 2010 in people not taking statins or blood pressure drugs.
2. The age specific relative risks of a stroke or myocardial infarction (MI) whilst on the polypill.
3. The age-specific mortality from all causes other than MIs or strokes in England and Wales 2010.

In order to be consistent with the earlier paper [8, 9], the same 2010 incidence and mortality rates were used. However, 2013 population figures were used, in order to have the most up to date estimates of the costs and benefits of a Polypill Prevention Programme.

## The incidence of first myocardial infarction (MI) or stroke in England and Wales in 2010 in people not taking statins or blood pressure drugs

To estimate this, we first used published estimates of the incidence of these disorders in 1985–1995 and then adjusted them for the reductions in incidence that occurred between 1995 and 2010, and then took account of the fact that about 30 % of people aged 50 and older were taking statins or blood pressure drugs in 2010.

### Annual incidence of first MI and stroke from 1985 to 1995

The following unpublished weighted logistic regression equations from the meta-analysis reported by Law et al. [38] were used to obtain yearly age specific incidence rates for men:

$$ \begin{aligned} & {\text{incidence}}\;{\text{of}}\;{\text{first}}\;{\text{MI}} = \exp \left( { - 8.9041 + 0.06148 \times {\text{years}}} \right)/\left( {1 + \exp \left( { - 8.9041 + 0.06148 \times {\text{years}}} \right)} \right),\;{\text{and}} \\ & {\text{incidence}}\;{\text{of}}\;{\text{first}}\;{\text{stroke}} = \exp \left( { - 11.3454 + 0.08769 \times {\text{years}}} \right)/\left( {1 + \exp \left( { - 11.3454 + 0.08769 \times {\text{years}}} \right)} \right). \\ \end{aligned} $$

For women:

$$ \begin{aligned} & {\text{incidence}}\;{\text{of}}\;{\text{first}}\;{\text{MI}} = \exp \left( { - 12.5712 + 0.10332 \times {\text{years}}} \right)/\left( {1 + \exp \left( { - 12.5712 + 0.10332 \times {\text{years}}} \right)} \right),\;{\text{and}} \\ & {\text{incidence}}\;{\text{of}}\;{\text{first}}\;{\text{stroke}} = \exp \left( { - 11.8133 + 0.09112 \times {\text{years}}} \right)/\left( {1 + \exp \left( { - 11.8133 + 0.09112 \times {\text{years}}} \right)} \right). \\ \end{aligned} $$

### Allowing for the decrease in incidence and case fatality from 1985–1995 and to 2010

The incidence estimates in the previous paragraph relate to 1985–1995. Since then mortality from MI and stroke has decreased [ONS 1985–1995 [39] vs ONS 2010 [40, 41] as shown in column 3 in Table 6], as a result of both a decrease in incidence and a decrease in case-fatality. Two studies [2, 42] reported the contributions to the decrease in mortality arising from a decrease in incidence (I) compared with a decrease in case fatality (CF) (column 4 in Table 6). The decrease in incidence of first MI and strokes from 1985–1995 to 2010 (column 5) was estimated using the results of these two studies and assuming I and CF changed, over time, by the same proportion (P) (so that the decrease in incidence is P × I and the decrease in case fatality is P × CF). Then the decrease in mortality is 1 − ((1 − P × I) (1 − P × CF)). For example in the second row of Table 6 the decrease in mortality is 67 %, so 0.67 = 1 − (1 − 0.30P)(1 − 0.43P) which can be rearranged so that 0.129P^2^ − 0.73P + 0.67 = 0. This quadratic equation has two solutions; P = 1.15 and P = 4.51. P = 4.51 leads to a decrease in incidence greater than 100 %, which is not possible, so the decrease in incidence, P × I = 1.15 × 30 % = 35 % (as given in col 5).

**Table 6:** Estimated decrease in incidence of MI and stroke from 1985–1995 based on mortality changes and the contributions from changes in incidence and case-fatality

Gender	Disorder	Observed decrease in mortality from 1985–1995 to 2010 (%)	Decrease in incidence; Decrease in case fatality [2, 42] (%)	Estimated decrease in incidence from 1985–1995 to 2010 (%)
Female	MI	76	31^a^; 29^a^	53
Female	Stroke	67	30^b^; 43^b^	35
Male	MI	69	33^a^; 24^a^	51
Male	Stroke	64	30^b^; 43^b^	33

^a^2002–2010

^b^1999–2008

The age specific incidence of first MI and stroke in 2010 was estimated by multiplying the estimated decreases in incidence from 1985–1995 to 2010 (column 5 in Table 6) by the logistic regression equations for the age specific incidence of first MI and stroke in 1985–1995 given in Annual incidence of first MI and stroke from 1985 to 1995.

### Allowing for the current use of components of the polypill in 2010

Around 30 % of people aged 50–99 were currently taking blood pressure lowering drugs [43] or statins [44] in 2010. Therefore the estimated age specific incidence in 2010 at each age was adjusted by 1/(0.7 + 0.3 × age specific relative risk as given in Table 7) to estimate the incidence in people not taking statins or blood pressure drugs. Details of the estimation of the age specific relative risks are given below.

**Table 7:** Age specific relative risk estimates

Age taking polypill	Relative risk of a first stroke on polypill^a^	Relative risk of a first MI on polypill^a^
50	0.26	0.13
60	0.31	0.23
70	0.38	0.33
80	0.51	0.37
90+	0.51	0.37

^a^Polypill contains simvastatin 20 mg, amlodipine 2.5 mg, losartan 25 mg, and hydrochlorothiazide 12.5 mg

## Estimating the age specific relative risks of a stroke or myocardial infarction (MI) on the polypill

Table 7 shows the age specific relative risk of a first MI or stroke based on four sources [3, 5, 6, 16]. The age specific relative risk estimates by single year of age for people aged 50–90 were obtained by linear interpolation using the relative risks in appendix Table 7. For people age 90 and above the relative risks were assumed to be constant.

## Estimating the age-sex-specific mortality and deaths from all causes other than an MI or stroke in England and Wales in 2010

Age-sex-specific mortality rates were obtained from the ONS publication Mortality Statistics: Deaths Registered in England and Wales (Series DR), 2010 [40]. The mortality rates from causes other than MI and stroke were calculated as the all-cause mortality rates minus the mortality rates from MI or stroke. These age-sex specific rates were applied to the number of people in the two 50 year old cohorts specified in the Methods (one taking the polypill and one not) to estimate the number of deaths occurring from causes other than MI or stroke each year. These were subtracted from the number of people in each cohort who were alive at the end of each year without having had an MI or stroke to reduce the size of the cohorts as they aged.

## Calculation of health service cost associated with the treatment of myocardial infarction

The total NHS healthcare cost for coronary heart disease used in this paper was £3460 million (2004 prices) [11]. This sum is taken from Table 2 in the reference cited [11] by subtracting the private healthcare cost of £399 million from total healthcare cost of £3859 million.

Coronary heart disease includes conditions related to myocardial infarction such as angina. The vast majority of coronary heart disease cost is myocardial infarction. Also, in expectation, the polypill will reduce incidence of coronary heart disease other than myocardial infarction such as angina so it is reasonable to use overall coronary heart disease cost in the calculation.

In 2006, there were an estimated 146,000 myocardial infarctions in the UK [15]. Therefore, the estimated coronary heart disease cost per myocardial infarction is £3460 million/146,000, or £23,699 (in 2004 prices). Taking into account inflation [13], current cost in 2014 was £29,900.

## Calculation of health service cost associated with the treatment of stroke

The total NHS healthcare cost for stroke has been estimated at £4384 million in 2005 (Table 2 in the reference cited) [12].

The same reference gives an estimate of 106,675 strokes a year (supplementary data of Saka et al. [12]). Therefore, the estimated cost per stroke is £4384 million/106,675, or £41,097 (in 2005 prices). Taking into account inflation [13], current cost in 2014 was £50,500.

## Calculating the costs separately the four countries in the UK

In order to calculate the total costs separately for each country in the UK the cohort analysis was repeated with the numbers of males and females aged 50 in 2013 in each country used instead of the number of males and females aged 50 in the UK (see Tables 8, 9).

**Table 8:** Numbers of males and females aged 50 in 2013 in the UK [10]

	Number of people aged 50 in 2013	
	Males	Females
England	379,474	388,016
Scotland	39,965	42,250
Wales	21,490	22,221
Northern Ireland	12,984	12,985
United Kingdom	453,913	465,472

**Table 9:** Total saving, total cost and net cost or saving each year in people aged 50 and over in a Polypill Prevention Programme according to the four countries in the UK in a “working case”

	Total saving from reducing incidence of MI and stroke (£bn)	Daily cost of polypill prevention programme per person (£)									
		0.50		0.75		1.00		1.25		1.50	
		Total cost of polypill prevention programme (£bn)	Net cost or saving (£bn)	Total cost of polypill prevention programme (£bn)	Net cost or saving (£bn)	Total cost of polypill prevention programme (£bn)	Net cost or saving (£bn)	Total cost of polypill prevention programme (£bn)	Net cost or saving (£bn)	Total cost of polypill prevention programme (£bn)	Net cost or saving (£bn)
	(1)	(2)	(2–1)	(3)	(3–1)	(4)	(4–1)	(5)	(5–1)	(6)	(6–1)
England	2.22	1.99	(0.23)	2.98	0.77	3.97	1.76	4.96	2.75	5.95	3.74
Scotland	0.24	0.21	(0.02)	0.32	0.08	0.43	0.19	0.53	0.29	0.64	0.40
Wales	0.13	0.11	(0.01)	0.17	0.04	0.23	0.10	0.28	0.16	0.34	0.21
Northern Ireland	0.07	0.07	(0.01)	0.10	0.03	0.13	0.06	0.17	0.09	0.20	0.13

*MI* myocardial infarction
